# Nanopulse Stimulation (NPS) Induces Tumor Ablation and Immunity in Orthotopic 4T1 Mouse Breast Cancer: A Review

**DOI:** 10.3390/cancers10040097

**Published:** 2018-03-30

**Authors:** Stephen J. Beebe, Brittany P. Lassiter, Siqi Guo

**Affiliations:** Frank Reidy Research Center for Bioelectrics, 4211 Monarch Ways, Suite 300, Norfolk, VA 23508, USA; blassite@odu.edu (B.P.L.); s2guo@odu.edu (S.G.)

**Keywords:** programmed cell death, immunogenic cell death, immunosuppression, tumor microenvironment, danger-associated molecular patterns, plasma membrane charging time constant (3–10)

## Abstract

Nanopulse Stimulation (NPS) eliminates mouse and rat tumor types in several different animal models. NPS induces protective, vaccine-like effects after ablation of orthotopic rat N1-S1 hepatocellular carcinoma. Here we review some general concepts of NPS in the context of studies with mouse metastatic 4T1 mammary cancer showing that the postablation, vaccine-like effect is initiated by dynamic, multilayered immune mechanisms. NPS eliminates primary 4T1 tumors by inducing immunogenic, caspase-independent programmed cell death (PCD). With lower electric fields, like those peripheral to the primary treatment zone, NPS can activate dendritic cells (DCs). The activation of DCs by dead/dying cells leads to increases in memory effector and central memory T-lymphocytes in the blood and spleen. NPS also eliminates immunosuppressive cells in the tumor microenvironment and blood. Finally, NPS treatment of 4T1 breast cancer exhibits an abscopal effect and largely prevents spontaneous metastases to distant organs. NPS with fast rise–fall times and pulse durations near the plasma membrane charging time constant, which exhibits transient, high-frequency components (1/time = Hz), induce responses from mitochondria, endoplasmic reticulum, and nucleus. Such effects may be responsible for release of danger-associated molecular patterns, including ATP, calreticulin, and high mobility group box 1 (HMBG1) from 4T1-Luc cells to induce immunogenic cell death (ICD). This likely leads to immunity and the vaccine-like response. In this way, NPS acts as a unique onco-immunotherapy providing distinct therapeutic advantages showing possible clinical utility for breast cancers as well as for other malignancies.

## 1. Introduction

Nanopulse Stimulation (NPS), which has previous been called nanosecond pulsed electric fields (nsPEFs), nanosecond electric pulses (nsEPs), and nanoelectroablation, has been shown over the last decade or more to eliminate mouse and rat tumor types in syngeneic and non-syngeneic models with orthotopic and ectopic localizations as well as with human xenograft models ([1], reviewed). NPS technology is based on pulsed power physics by accumulating electric energy and releasing it in nanosecond bursts, which produces high-power and low-energy electric pulses. While conventional electroporation (EP) uses a similar concept, the power equivalent for NPS is 3 orders of magnitude less comparing a 10 µs (0.1 MW) with a 10 ns (100 MW) pulse. In addition to this power domain, NPS is most often delivered with duration near the plasma membrane charging time constant (~75 ns). When considered in the frequency domain (1/time = Hz), such pulses exhibit transient, high-frequency components with fast rise–fall times. Thus, in the frequency spectrum the 10 ns (100 MHz) pulse displays frequencies 3 orders of magnitude greater than does a 10 µs pulse (0.1 MHz). NPS pulses are also different because when applied with relatively low repetition rates (1–5 Hz), NPS is nonthermal [2,3]. While these nanosecond pulses deliver high electric fields with tens of kV/cm, they are safe and essentially free of local and systemic side effects. After animals are treated with NPS and recover from anesthesia, they quickly return to normal activity. During treatment, there are no muscle contractions, so paralytic agents are not necessary. Although some scab formation occurs on the stratum corneum in ectopic models (and the orthotopic mammary model), this is resolved within two weeks as the stratum corneum is regenerated [2]. In a clinical trial with human skin, physical effects on collagen and elastic fibers in the fiber septum are evident and minor dermal injury resulted in inflammation and pruritus, but this was resolved within two weeks, leaving no scar or skin discoloration [4]. Thus, NPS is a safe procedure in spite of the high electric fields. When 1000 pulses are applied at 50 kV/cm, the total time tumors are exposed to 100 ns pulses is only 0.1 milliseconds. This brief exposure to NPS is sufficient to not only eliminate tumors, but also to induce an immune response.

## 2. Evidence That NPS Induces Immune Responses

In essentially all studies using NPS for cancer treatment, it is possible to eliminate high percentages of tumors (75–100%) when electric field intensity and pulse numbers are sufficient. In this study and others recently carried out in the Center for Bioelectrics [5,6,7], 800–1000 pulses at 1–3 Hz with durations of 100 ns and electric field strengths of 50 kV/cm are sufficient to eliminate essentially all tumors. When fewer pulses (300–500) are used under these conditions [6] or when 30 ns pulses are used with electric field strengths as high as 68 kV/cm [5], tumor elimination is not complete. It is likely for pulses as short as 30 ns that much higher electric field intensities are needed for tumor elimination. This follows the requirement that as pulse durations decrease, higher electric field strengths are needed for similar effects. Greater cell number can also compensate to some degree. Nevertheless, during and after sufficient NPS treatment times, profound changes take place in the tumor microenvironment (TME). Over the next 3–7 weeks after tumor ablation, events occur that essentially vaccinate rats against N1-S1 hepatocellular carcinoma HCC [6] and mice against 4T1 breast cancer [7]. When these animals are orthotopically challenged after ablation, respective tumors do not form. It was highly likely that these protective, vaccine-like effects were immune mediated. In UV-induced murine melanomas, NPS was better than tumor resection at hastening secondary tumor rejection, suggesting an immune response [8]. In orthotopic rat HCC and mouse fibrosarcoma allografts, NPS induced CD8-dependent attenuation of secondary tumor growth in rats depleted of CD8+ T cells. In addition, it was possible to vaccinate mice with NPS isogenic tumor cells and inhibit growth of secondary tumors in a CD8+-dependent manner, again suggesting that NPS induced immune responses [9]. In the present report, we review studies in the metastatic mouse 4T1-Luc breast cancer model that clearly and directly demonstrate multiple immune mechanisms that are responsible for this vaccine-like effect in mice [7]. While briefly previewed here, very similar mechanisms are operative in the rat HCC model [10].

## 3. Relationships between Programmed Cell Death (PCD) and Immunogenic Cell Death (ICD)

NPS inflicts significant damage to the TME in a way that tumor cells, as well as substantial numbers of host somatic and immune cells in the treatment zone, are sufficiently stressed such that they are unable to recover normal functions. They then undergo PCD as default mechanism(s) depending on the cell type, functional contexts, and intensity of the stimulus. NPS has been shown to induce apoptosis in several cell types in vitro [11,12,13,14,15,16] and in vivo [5,6,16]. However, NPS has also been shown to induce other PCD mechanisms such as parthanatos in HeLa S3 cells [15]. Whether these cells have an apoptotic cell death (CD) pathway blocked or whether this is the natural PCD mechanism for these cells in response to NPS has not been investigated. It has also been shown that nonlethal or sub-toxic NPS induces autophagy, likely as a mechanism to salvage tissue and repair membrane and organelle damage [17]. However, when NPS was more severe, this compensatory mechanism fails and PCD was induced. Autophagy, or self-eating, is a conserved mechanism to maintain cellular homeostasis by degrading cellular constituents that are defective, such as organelles and/or misfolded proteins [18,19]. Autophagic mechanisms are upregulated during stresses such as NPS, initially, to sustain cell functions by recycling cellular components as alternative energy sources. Whether autophagy primarily serves solely to support survival in response to cell stress and/or to induce CD mechanisms itself is a considerable controversy [18]. One approach for autophagy analysis is to induce cell stresses and then block a CD mechanism such as apoptosis, for example [18]. This is analogous to a cancer hallmark where apoptotic CD is blocked as a mechanism of cancer. When sufficiently stressed, cells will die by one mechanism or another, and depending on the physiological/pathological context, post-autophagic CD is likely cell-type- and cell-context-specific. Even within the same cell type, NPS-induced CD can be caspase-dependent or caspase-independent depending on the NPS conditions [13,20].

It is now generally believed that specific mechanisms induced during CD are critical for an immune response. However, there is debate about whether apoptosis, necroptosis, necrosis, and/or autophagy are important for ICD induction. It is possible, depending on the stimulus and its intensity, that more than one CD program can be functioning simultaneously. During developmental and homeostatic CD, apoptosis is anti-inflammatory and immunologically silent or tolerogenic [21]. However, a number of recent studies indicate that caspase-dependent processes are also important for immunogenicity [22]. In chemotherapy-induced CD, some (anthracyclins), but not all (mitomycin C) caspase-inducing chemotherapeutic agents initiate ICD [23,24]; there are immunogenic and non-immunogenic subcategories of apoptosis that have yet to be specifically differentiated. Apoptosis has been shown to induce maturation of dendritic cells (DCs) leading to T cell activation and immunity [25] and that apoptotic cells not only undergo degradation, but also deliver processed antigens to DCs for cross-presentation [24]. However, while DCs are able to distinguish two types of tumor apoptotic CD, programmed necrosis mechanisms may also be able to provide a control that is critical for the initiation of immunity [26,27]. Autophagy and the release of ATP may also to be required for ICD [28,29].

Immunogenic cell death has obvious advantages for cancer treatment, but little is known about how CD pathways influence these immune mechanisms. In the last several years, it has been suggested that there is a relatively specific set of effector molecules that play critical roles in ICD. These are generally referred to as damage-associated molecular patterns (DAMPs) and are recognized by pattern recognition receptors (PRRs) on antigen-presenting cells (APCs). They include changes in cell surface membranes (externalized calreticulin binds to CD91 on DCs enhancing engulfment) [30,31] and release of soluble factors that interact with a series of DC receptors to enhance antigen presentation to T cells high mobility group box 1 protein (HMGB1) binds toll-like receptors and ATP binds to purinergic P2RX7 stimulating IL-1β). Cancer cells also release “stress ligands” that are recognized by immune cells. These events lead to activation of the immune system against cancer [22]. Thus, increasing evidence indicates that certain mechanisms of tumor CD can enhance immune responses through ICD. However, whether immunogenicity depends on regulated cell death (RCD) by apoptosis, necrosis, autophagy, or all of them [32] remains to be determined.

**Figure 1:**
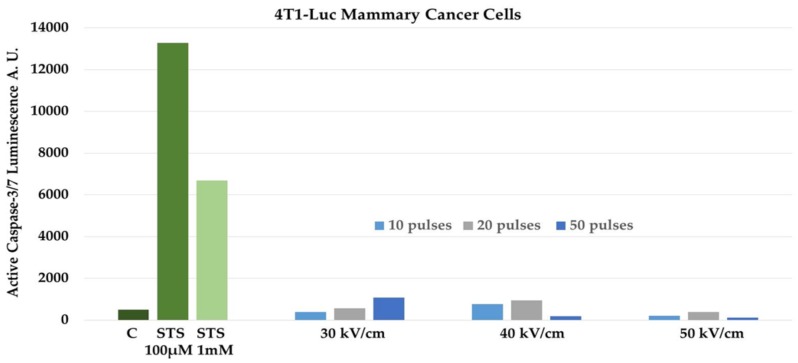
Staurosporine (STS), but not Nanopulse Stimulation (NPS), induces caspase-3/7 activity in 4T1-Luc mammary cancer cells. 4T1-Luc cells were treated with 100 µM and 1 mM STS or treated with 10, 20, and 50 pulses with 60 ns pulse durations and electric fields of 30, 40, and 50 kV/cm. Control (C) cells were untreated with STS or NPS. Five (5) hours later, caspase-3/7 activity was determined using the Caspase-Glo assay (ProMega, Madison WI) according to the supplier’s recommendation. The figure represents a typical assay with triplicate determinations.

Recent evidence proposes that during PCD externalization of calreticulin (CRT) from the endoplasmic reticulum, release of HMGB1 from the nucleus and release of ATP have an expressive impact on the immune response [33]. All of these DAMPs have been shown to be released from cells dying after NPS [7,16]. Apoptotic Jurkat cells were shown to express calreticulin on their cell membranes and release ATP and HMGB1 after exposure to NPS at levels that were similar to those released by doxorubicin and mitoxantrone, two known inducers of ICD [16]. NPS-treated 4T1 cells were shown to release DAMPs including calreticulin, HMGB1, and ATP [7]. It was of interest to determine if NPS induced apoptosis in 4T1-Luc cells. Since NPS has been shown to induce apoptosis in several cell types in vitro [11,12,13,14,15,16] and in vivo [5,6], we used a well-defined catalytic caspase assay to determine if NPS induced apoptosis in 4T1-Luc cells. We used staurosporine (STS) as a positive control. Staurosporine is well known to induce caspase-dependent apoptosis in a number of cell types. The mechanism of STS-induced apoptosis is controversial. It has been shown to increase intracellular Ca^2+^, elevate reactive oxygen species (ROS) and peroxidate proteins and lipids [34], activate c-Jun N-terminal kinases (JNK) and induce transcription through AP-1 and NFκB [35], and to inhibit kinase activities by competing with ATP binding [36]. However, what is known is that one or more of these mechanisms results in caspase activation to induce apoptosis. Figure 1 shows that when 4T1-Luc cells were treated with 100 µM and 1 mM staurosporine, caspase-3/7 catalytic activity was readily induced compared with in untreated control cells. Treatment with 1 mM STS caused less caspase-3/7 activity than with 100 µM, most likely due to excessive loss of cells before caspase activity was determined. In contrast, NPS had very little effect on caspase-3/7 activity when treated with 10, 20, or 50 pulses with durations of 60 ns and electric field intensities of 30, 40, or 50 kV/cm. At higher electric fields and/or greater pulse numbers, caspase-3/7 activity actually decreases compared with at lower conditions. Again, this is most likely due to loss of cell numbers before assay at higher treatment conditions.

There are a number of cancer-regulated mechanisms that can prevent PCD such as increased expression of anti-apoptotic Bcl-2 proteins to prevent mitochondrial outer membrane permeabilization, expression of caspase inhibitors such as X-linked inhibitor of apoptosis protein (XIAP) or cytokine response modifier A (CrmA), mutations in proteins such as p53, ATM, Rb, or PTEN, and dysregulation of NFκB, Myc, Ras, or Akt, among many others [37,38]. Nevertheless, when 4T1 tumors are treated with NPS in vivo, they induce T cell memory responses that protect against recurrence, exhibit an abscopal effect, and prevent metastasis to distant organs, as will be discussed below. Given that Jurkat cells [13,16] and 4T1 breast cancer cells (Figure 1) undergo different CD mechanisms, yet both release DAMPs implicated in ICD, it is possible that NPS-induced release of ICD-inducing DAMPs may be independent of a specific CD mechanism(s). Alternatively, different tumor structural features may affect regional electric field intensities, or heterogeneity of electric field intensities within the treatment zone may induce heterogeneous PCD mechanisms, one or more of which is immunogenic. The expression of DAMPS by NPS in 4T1 cells, which do not undergo NPS-induced caspase activation in vitro, correlated with ICD of 4T1 tumors in vivo.

## 4. Does the Plasma Membrane Charging Time Constant (or High-Frequency Components) Determine NPS-Induced Immune Mechanisms

It is particularly noteworthy that all of these ICD signals arise from intracellular structures. It is likely not fortuitous that NPS induction of ICD is related to its unique electric field characteristic to induce intracellular effects. This was first shown when intracellular vesicles of eosinophils treated with NPS were permeabilized by NPS and cytosolic, anionic calcein entered breached intracellular vesicles containing cationic proteins inducing “sparkler morphology” [39]. These intracellular effects were hypothesized to be due to NPS that was applied in the same time domain (60 ns in that study) as the plasma membrane charging time constant, which is on the order of 75 ns [39]. This concept can also be understood from a different perspective by considering pulse durations in the frequency domain (1/s = Hz) instead of the time domain. The hypothesis states that it is the rapidly changing electric fields during the rise and fall of the pulse amplitudes, or the transient features, that coincide with intracellular effects. Monopolar square-wave pulses that have durations near the charging time of plasma membranes naturally exhibit rapid rise and fall times [39,40]. So, these transient high-frequency components from rapid rise–fall times reach beyond the plasma membrane to intracellular structures, while plasma membrane effects occur primarily at the pulse plateau.

The importance of the rise–fall times for NPS effects was demonstrated by showing that 600 ns pulses with fast rise–fall times (15 ns) had greater effects in dissipating mitochondrial membrane potential (ΔΨm) and inducing CD than did 600 ns pulses with slow rise–fall times (150 ns) [20,40]. The rise–fall times did not affect Ca^2+^ influx through the plasma membrane, so these rise–fall time effects were selective for intracellular structures, as hypothesized. The effect on ΔΨm was closely correlated with loss of cell viability. When pulse waveforms induced calcium influx without effects on ΔΨm, there were no effects on viability, implicating loss of ΔΨm with loss of cell viability [40,41]. Consequently, these intracellular effects do have functional consequences. It was also shown that the high-frequency components of the fast rise–fall times affected the ΔΨm in a calcium-dependent manner. The Ca^2+^ dependence suggests that these effects are independent of membrane pore formation.

In other studies analyzing Ca^2+^ mobilization, pulses with shorter durations—which have higher-frequency components—had greater effects on Ca^2+^ release from the intracellular stores than did longer pulses {10 ns (100 MHz) > 60 ns (16.7 MHz) > 300 ns (3.3 MHz)} [42]. Therefore, these NPS-induced intracellular effects apply to intracellular calcium stores, most likely the endoplasmic reticulum (ER). This suggests possible roles for NPS to stimulate intracellular Ca^2+^ signaling by porating to ER.

NPS intracellular effects were also shown in the nucleus. In Jurkat cells, NPS with 10 ns duration affected nuclear speckles, also known as small nuclear ribonucleoprotein particles (snRNPs) or intrachromatin granule clusters (IGCs) [43]. When Jurkat cells synchronized in the mitotic (M) phase (95%) were treated with a single 10 ns, 150 kV/cm pulse, snRNPs decreased immediately following exposure, but then increased 3 h later compared with in the unpulsed cells, indicating functional and conformational changes in intranuclear subunits. This could have caused at first a decrease and then a compensatory increase in RNA processing. When cells in the M phase were treated with 5 consecutive, 10 ns pulses, the numbers of snRNPs immediately increased. This indicates that NPS can also affect RNA-protein nuclear substructure complexes and possibly RNA processing.

Although it has not been tested fully, based on these data, it is likely that NPS conditions with pulse durations with fast rise–fall times near the plasma membrane charging time constant have specific effects on the mitochondria, ER, and/or the nucleus that have physiological and pathological consequences depending on the stimulus intensity. It is then not surprising that fast rise–fall times under intense NPS conditions could induce translocation of calreticulin from the ER to the plasma membrane, affect nuclear substructures that contain HMGB1, and affect ΔΨm by opening of the Ca^2+^-dependent mitochondrial permeability transition pore (mPTP) to induce an ICD.

Given that mitochondria control CD mechanisms through effects on the mPTP and it is proposed that NPS affects the mPTP, a closer look at NPS effects on ΔΨm are germane. The calcium dependence of NPS-induced loss of ΔΨm suggests effects on the mPTP, which depends on Ca^2+^ for opening. While there has been a considerable debate for years about what comprises the mPTP and what constitutes the pore itself, the most recent hypothesis is that the mPTP is a dimer of ATP synthase, complex V of the electron transport chain, with the dimeric interface forming the pore itself [44]. What has not been debatable during this controversy is that the mPTP is regulated by cyclophilin D, which is activated to open the mPTP in a calcium-dependent manner. A hypothetical mechanism is that NPS affects a Ca^2+^-sensitive site on cyclophilin D as a component of the ATP synthase causing opening of the mPTP and loss of ΔΨm leading to PCD. Whether this initiates CD by apoptosis, some other PCD mechanisms, or unprogrammed necrosis depends on a number of factors such as the stimulus, stimulus intensity, the cell and its physiology/pathology, and other contextual phenomena. Additional studies are required to determine the NPS-induced 4T1 CD mechanism(s) that addresses this hypothesis; however, it will likely be found, at least in part, within mitochondria. Since Ca^2+^ primarily exerts effects on proteins and NPS affects Ca^2+^-dependent effects on ΔΨm, this further suggests that NPS can affect proteins [41] or protein complexes such as cyclophilin D and/or snRNP in IGCs [43], both of which can affect higher-order molecular consequences.

## 5. NPS Targets the Tumor Microenvironment (TME)

From the earliest approaches for cancer therapy, the neoplastic cells themselves have been the obvious focal target. From here, the hallmarks of cancer were defined [45,46], oncogenes and suppressor genes were discovered, cancer signaling mechanisms were delineated, and cancers were identified at base pair resolutions [47]. Now, whole-gene sequencing, transcription profiling, and epigenetic analyses have provided a deeper understanding of cancer cell transformations [48]. However, it has been known for some time that malignant transformation and cancer progression cannot occur without contributions from a number of host cell types that subsidize the angiogenic switch, epithelial–mesenchymal transformation, and invasion/metastasis, among other cancer mechanisms. So, recognition of the importance of the TME in the evolution of cancer has refocused attention to a more complex landscape defining cancer in a much broader context than just tumor cells themselves. One of the most confounding features for immunotherapy is the immunosuppressive behaviors of T regulatory cells (Tregs) and myeloid-derived suppressor cells (MDSC) in the TME.

Immuno-inhibitory cytokines such as IL-10, IL-35, and TGFβ are secreted by Tregs that interact with DCs in the TME and inhibit T cell effector functions, induce T cell anergy, prevent T and B cell proliferation, and inhibit antitumor functions of natural killer cells (NKs), DCs, and macrophages [49]. The TME promotes epithelial–mesenchymal transition, drug resistances, and angiogenesis, which promotes metastasis [50,51]. Without NPS treatment, the immunosuppressive TME of 4T1 mammary cancer and other tumors prevents effective innate and adaptive immune responses requiring humane euthanasia 2–3 weeks after tumor initiation due to tumor burden [6,7]. The alleviation of immunosuppression in the TME has far-reaching consequences beyond the primary NPS target site, including immunity at sites distant from the primary tumor.

While the primary focus of the NPS here is generally tumor cells, there is much more at stake to ablate cancer and induce an effective immune response in the TME. The tumor essentially develops into a rogue, parasitic organ that no longer provides functional benefits to the host. In addition to tumor cells, the TME also includes stromal cells from the patient, including somatic cells, endothelial cells, and cohorts of suppressor Tregs and MDSC that thwart immune functions and also interact with cancer stem cells that promote tumorigenesis, drug resistances, and metastasis [52]. In addition, this inhibits effector DC function as APCs in the TME, now unable to retrieve antigens effectively from dead and dying tumor cells as a means to promote adaptive memory responses against the tumor. NPS elimination of tumor cells, tumor stroma, vascular endothelia cells, Tregs, and MDSC disrupts these tumor-promoting collaborations in this hostile TME. In 4T1-Luc tumor-bearing mice, Tregs and MDSC were elevated eight- to tenfold in blood. After NPS, overexpression of Tregs and MDSC numbers decrease not only in the TME, but also in blood, providing a possible marker for therapeutic efficacy of NPS [7].

## 6. NPS Promotes Activation of DCs

**Figure 2:**
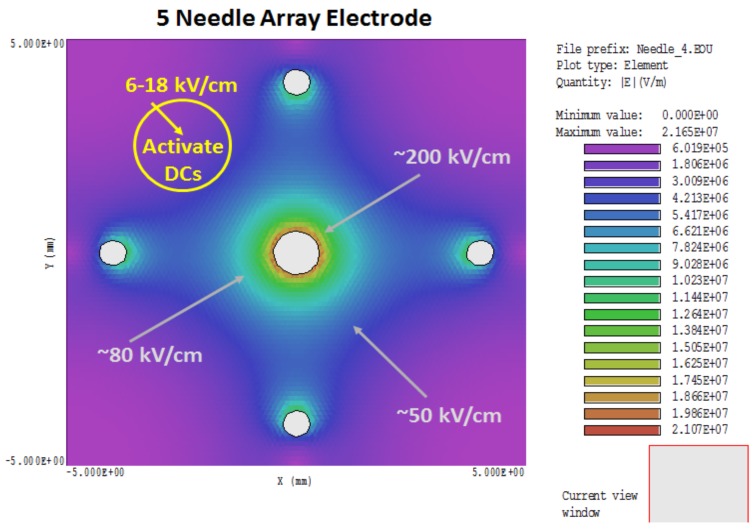
NPS electric field simulation: Electric fields were simulated from an NPS treatment zone of 50 kV/cm (25 kV across 0.5 cm) using a 5-needle electrode array. The electric field is heterogeneous with very high electric fields near the needles and lower, nonlethal electric fields in the periphery where DCs can be activated by NPS.

NPS can be a two-sided coin. In addition to tumor cell ablation, it has been previously proposed and shown that NPS can bypass receptors and induce effects without ligands [53]. One early example showed that NPS activated platelets, effectively serving as proxy to bovine thrombin [54]. A more recent example is very low, nonlethal NPS-induced activation of mouse bone-marrow-derived (BMD) DCs, defined as increases in cell surface expression of MHCII, CD86, and CD40 [7]. Figure 2 shows the heterogeneous NPS treatment zone with the 5-needle array electrode. The electric field around the needles themselves (80–200 kV/cm) is considerably higher than the so-called electric field of 50 kV/cm, which is determined as the voltage divided by distances between electrodes (25 kV across 0.5 cm, in this case). However, the electric fields decrease in areas away from the needles, such that in regions equidistant between needles, electric fields are closer to that defined by voltage/distance between needles. In regions outside the electrode array, electric fields are not lethal, but could be stimulatory to cells in this region. This suggests that in zones adjacent to an NPS-lethal tumor target, electric fields with much lower amplitudes can activate DCs [7], as well as macrophages [Bani Hani and Beebe, unpublished]. NPS-induced activation of DCs mimics DC expression of activation receptors when they are incubated with 4T1 cells that were treated with NPS [7]. This possible activation of DCs outside the lethal NPS zone adjacent to regions where the TME is collapsing and disintegrating is much less suppressive in the absence of Tregs and MDSC [7] and more favorable for entering and remodeling the old TME and identifying cancer antigens. The immune response starts in this reconfiguring TME, where the immunosuppression is dissipating, tumor cells are dying with their proliferative signaling quenched, and ICD signaling, at least in part, intact and functional after NPS. Antigens are identified here and cross-presented to lymphocytes in lymph nodes.

Given the heterogeneity of the electric field in the 5-needle array treatment zone, with electric fields ranging from 20 to 200 kV/cm, it is probable that cells within these different zones are dying by different PCD mechanisms depending on the electric field intensity. Some cells may be undergoing nonprogrammed necrosis around the needles, especially the center needle, while others may be going through different PCD between the needles, and yet others may be experiencing autophagy before executing yet another PCD mechanism on the outer reaches of the treatment zone. This is also likely applicable to a two-needle electrode [9], except the heterogeneity is distributed differently. This concept is a corollary to the possibility that NPS-induced release of DAMPs causing ICD may be independent of a specific CD mechanism. Otherwise, in the 2- and 5-needle electrode designs, the electric field heterogeneity may ensure that one or more PCD mechanisms is adequate to induce ICD mechanisms.

## 7. NPS Activates Immune Memory

While lethal NPS induces PCD that promotes expression of ICD-related DAMPs; lethal NPS terminates immunosuppressive constituents in the TME, including Tregs and MDSC; and NPS activates DCs and macrophages in adjacent nonlethal regions of the treatment zone, which enhances their antigen presenting functions; T-lymphocytes are taught to remember these infringing tumor cells to participate in tumor elimination and to remove them should they return. In the 4T1 breast cancer model, all 11 mice whose tumors were eliminated by NPS were resistant to challenge injections of 4T1 cells. CD4+ effector memory (Tem, CD44+ CD62L−) and CD8+ Tem and central memory (Tcm, CD44+ CD62L+) T-lymphocytes were elevated in spleen and blood after NPS. Both CD4+ and CD8+ T-lymphocytes were cytotoxic, releasing increased levels of IFNγ [7]. This demonstrates a potent antitumor immune memory response due to NPS of mouse 4T1 mammary cancer. These mice have essentially been vaccinated by the treatment. It was also shown that MCA205 fibrosarcoma cells treated with NPS (100 ns, 50 kV/cm, 500 pulses) vaccinated mice against challenge injections of the same cells [9].

## 8. NPS Reduces Spontaneous Metastasis

NPS is primarily a local ablation therapy. However, the presence of an immune response suggests that there may be more to this therapy than ablation and prevention of recurrence. This depends on how robust the immune response is. The 4T1/4T1-Luc breast cancer model is well known to be poorly immunogenic with high spontaneous metastasis to distant organs [55,56] like that found in humans [56,57]. The 4T1-Luc model used here expresses a luciferase reporter gene that is activated when provided with its substrate luciferin, so it is possible to monitor expiration of the primary tumor as well as determine the presence of distant metastasis. In a study using 600 pulses with durations of 100 ns and electric fields at 50 kV/cm, not all primary tumors were eliminated. When metastasis was analyzed in NPS-treated tumors and control tumors of the same size, 82% (9/11) of control mice exhibited metastasis to the liver, lungs, and spleen, while only 14% (1/7) showed a small growth in the lung [7]. This it is especially noteworthy given that NPS induces immunity and inhibits metastasis from the primary site in this poorly immunogenic, very mutagenic, and highly metastatic mouse breast cancer model.

## 9. Conclusions

The study by Guo and colleagues [7], provides the first direct evidence that NPS initiates an immune response, which is responsible for the vaccine-like effect seen in the 4T1 model. The immune response is dynamic and relatively strong, eliminating tumor cells that release DAMPs to induce ICD in the TME, abolishing immunosuppressive Tregs and MDSC in TME and blood, activating adaptive immune memory, avoiding recurrence, and, most impressively, largely preventing distant metastasis. It will be interesting to define immune mechanisms after challenge of the protected animal. Most importantly, it will be important to show that immune responses occur in humans after NPS like those seen in rats and mice. One approach to do this, short of treating tumors in humans, is under way in a humanized mouse model that bears a well-characterized human triple-negative breast tumor and a human immune system established from human peripheral blood mononuclear cells.
